# Characteristics of Hyaluronan Synthesis Inhibition by 4-Methylumbelliferone in Orbital Fibroblasts

**DOI:** 10.1167/iovs.61.2.27

**Published:** 2020-02-21

**Authors:** Erika Galgoczi, Florence Jeney, Monika Katko, Annamaria Erdei, Annamaria Gazdag, Livia Sira, Miklos Bodor, Eszter Berta, Bernadett Ujhelyi, Zita Steiber, Ferenc Gyory, Endre V. Nagy

**Affiliations:** 1 Division of Endocrinology, Department of Medicine, Faculty of Medicine, University of Debrecen, Debrecen, Hungary; 2 Department of Ophthalmology, Faculty of Medicine, University of Debrecen, Debrecen, Hungary; 3 Department of Surgery, Faculty of Medicine, University of Debrecen, Debrecen, Hungary

**Keywords:** Graves’ orbitopathy, hyaluronan, 4-methylumbelliferone, hyaluronan synthase, hyaluronidase

## Abstract

**Purpose:**

Hyaluronan (HA) overproduction by orbital fibroblasts (OFs) is a major factor in the pathogenesis of Graves’ orbitopathy (GO). 4-methylumbelliferone (4-MU) is an inhibitor of HA synthesis in different cell types in vitro and has beneficial effects in animal models of autoimmune diseases.

**Methods:**

HA production and mRNA expression of HA synthases (*HAS1*, *HAS2*, and *HAS3*) and hyaluronidases (*HYAL1* and *HYAL2*) were measured in the presence and absence of 4-MU in unstimulated and transforming growth factor–β–stimulated fibroblasts from GO orbital (n = 4), non-GO orbital (n = 4), and dermal origin (n = 4).

**Results:**

The 4-MU treatment (1 mM) for 24 hours resulted in an average 87% reduction (*P* < 0.001) of HA synthesis, decreased the expression of the dominant HAS isoform (*HAS2*) by 80% (*P* < 0.0001), and increased the *HYAL2* expression by 2.5-fold (*P* < 0.001) in control OFs, GO OFs, and dermal fibroblasts (DFs) regardless of the origin of the cells. The proliferation rate of all studied cell lines was reduced to an average 16% by 4-MU (*P* < 0.0001) without any effects on cell viability. HA production stimulated by transforming growth factor–β was decreased by 4-MU via inhibition of stimulated *HAS1* expression in addition to the observed effects of 4-MU in unstimulated cases. Characteristics of HA synthesis inhibition by 4-MU did not differ in OFs compared with DFs.

**Conclusions:**

4-MU has been found to inhibit the HA synthesis and the proliferation rate in OFs in vitro, adding it to the list of putative therapeutic agents in a disease the cure of which is largely unresolved.

Graves’ orbitopathy (GO) is an autoimmune disease of the orbits that may accompany Graves’ disease.^1^ Immune cell infiltration of the orbital tissue and local cytokine release result in increased production of extracellular matrix glycosaminoglycans, mainly hyaluronan (HA) by orbital fibroblasts (OFs), and extracellular matrix remodeling.^2^^,^^3^ HA, because of its highly hydrophilic nature,^4^ binds a large amount of water; edematic swelling of orbital tissues is a known factor in the pathogenesis of GO. The obvious incongruence between the limited volume of the bony orbit and the expanded orbital content results in the protrusion of the eye. HA is not a passive matrix component; it regulates cell-cell and cell-matrix adhesion, facilitates cell-cell signaling, influences the migration of immune cells, and improves proliferation.^5^^–^^8^

HA is a linear polymer of repeating units of glucuronic acid (GlcUA) and N-acetyl-glucosamine synthesized by 3 different HA synthase enzymes (HAS1, HAS2, and HAS3).^9^ HA synthesis is regulated by various growth factors including transforming growth factor–β (TGF-β), which has been found in the orbital tissue of patients with GO and correlated positively with the clinical activity of the disease.^10^ We recently demonstrated that TGF-β enhances HA synthesis of OFs via stimulating *HAS1* expression.^11^ The breakdown of HA is carried out by 2 isoforms of hyaluronidases, HYAL1 and HYAL2.^12^

4-methylumbelliferone (4-MU) is a coumarin derivative with no anticoagulant property and is available as a spasmolytic over-the-counter drug in several European countries. It is a specific inhibitor of the HA synthesis in multiple cell lines, including fibroblasts, keratinocytes, melanoma, and pancreatic cancer cells.^7^^,^^13^^–^^16^ 4-MU is a substrate for uridine 5′-diphospho-glucuronyltransferases (UDP-glucuronyltransferases), and the glucuronidation of 4-MU to form 4-methylumbelliferyl glucuronide (4-MUG) depletes UDP-GlcUA, one of the substrates of HAS enzymes thus limits HA synthesis.^17^ In addition, 4-MU decreases the mRNA expression of HAS enzymes.^18^

The 4-MU treatment was beneficial in animal models of autoimmune diseases,^16^^,^^19^^,^^20^ but its potential role as a therapeutic agent in GO has not been raised before.

Here we show that 4-MU is an effective inhibitor of HA synthesis in OF in vitro both in unstimulated and TGF-β–stimulated cells; changes of the mRNA levels of the 3 isoforms of HAS enzymes and the 2 isoforms of HYAL enzymes, as well as proliferation rate of the cells after 4-MU treatment have also been studied.

## Materials and Methods

### Materials

M199 with Earles’ salts without L-Glumatine, penicillin/streptomycin, Glutamine Stable were purchased from Biosera (Nuaille, France), Dulbecco's phosphate-buffered saline solution without calcium and magnesium, trypsin–EDTA solution, freezing medium, TrypLE Express, fetal bovine serum (FBS) and TGF-β were purchased from Thermo Fisher Scientific (Waltham, MA, USA). 4-MU and 4-MUG were purchased from Sigma Aldrich (St. Louis, MO, USA). TRI reagent solution was purchased from Molecular Research Center, Inc. (Cincinnati, OH, USA).

### Tissue Samples

Orbital connective tissues were obtained from patients undergoing orbital decompression surgery for GO. Control normal orbital tissues were obtained during surgery for nonorbital eye diseases. Control dermal connective tissues were obtained during abdominal hernia operations from patients with no history of thyroid diseases. The characteristics of patients are shown in the Table.

**Table.** Characteristics of Patients from Whom the Tissues were Obtained

Patients	Age (y)	Sex	Disease	GD Duration from Onset to ODS (y)	GD Treatment^*^	GO Duration from Onset to ODS (y)	GO Treatment^†^	CAS at the Time of Surgery	Type of Surgery	Tissue Origin
1	37	F	GO	11	RI, thiamazole	8	Orbital irradiation, corticosteroid	1	ODS	Extraconal posterior OAT
2	42	F	GO	4	Thyroidectomy, thiamazole	2	Corticosteroid	1	ODS	Extraconal posterior OAT
3	44	F	GO	9	Thyroidectomy, PTU	1	Orbital irradiation, corticosteroid, pentoxiphylline	2	ODS	Extraconal posterior OAT
4	49	M	GO	3	RI	3	Corticosteroid	3	ODS	Extraconal posterior OAT

5	46	M	IO malignant melanoma^‡^^,^^§^	n.a.	n.a.	n.a.	n.a.	n.a.	Enucleation	Intraconal posterior OAT
6	66	M	IO malignant melanoma^‡^^,^^§^	n.a.	n.a.	n.a.	n.a.	n.a.	Enucleation	Intraconal posterior OAT
7	69	M	IO malignant melanoma^‡^^,^^§^	n.a.	n.a.	n.a.	n.a.	n.a.	Enucleation	Intraconal posterior OAT
8	71	M	IO malignant melanoma^‡^^,^^§^	n.a.	n.a.	n.a.	n.a.	n.a.	Enucleation	Intraconal posterior OAT

9	20	M	Abdominal hernia^§^	n.a.	n.a.	n.a.	n.a.	n.a.	Hernia surgery	Abdominal dermal tissue
10	52	M	Abdominal hernia^§^	n.a.	n.a.	n.a.	n.a.	n.a.	Hernia surgery	Abdominal dermal tissue
11	63	F	Abdominal hernia^§^	n.a.	n.a.	n.a.	n.a.	n.a.	Hernia surgery	Abdominal dermal tissue
12	78	M	Abdominal hernia^§^	n.a.	n.a.	n.a.	n.a.	n.a.	Hernia surgery	Abdominal dermal tissue

IO: Intraocular, CAS: Clinical Activity Score, ODS: Orbital decompression surgery, RI: Radioactive iodine, PTU: propylthiouracil, OAT: Orbital adipose tissue

* At the time of orbital surgery patients had suppressed TSH levels and high-normal thyroid hormone levels.

† During the last 2 months before orbital surgery, patients used only diuretics, β-blockers, thyroxine supplementation, and local measures.

‡ No patients had scleral invasion or orbital involvement of the tumor.

§ Patients had no other diseases, especially no history of thyroid disease.

The study was approved by the Regional and Institutional Ethics Committee of the University of Debrecen. The study was carried out in accordance with the Declaration of Helsinki. Consent has been obtained from each patient after full explanation of the purpose and nature of all procedures used.

### Cell Cultures

Human fibroblasts were cultured as described previously in detail.^11^ The cells were studied between passages 2 and 8. For experiments, orbital and dermal fibroblasts (OFs and DFs) were plated in 24-well or 96-well plates, in confluent cell density (1.56 × 10^4^ cells/cm^2^) in M199 supplemented with 10% (v/v) FBS. The cultures were synchronized with serum starvation for 24 hours followed by treatment with medium containing 10% (v/v) FBS with or without 4-MU (0.125–6 mM) and TGF-β (1 ng/mL) or 4-MUG (1 mM) for an additional 24 hours. The 4-MU and 4-MUG stock solutions were dissolved in dimethyl sulfoxide (500 mM). The final concentration of dimethyl sulfoxide in the medium was adjusted to 0.2% in all experiments. All experiments were performed at least 3 times and carried out in triplicate. Secreted HA levels in cell culture supernatants and levels of pericellular HA^11^ were measured using the DuoSet Hyaluronan Kit (R&D Systems, Minneapolis, MN, USA)*.* In each case, results were adjusted for the HA content of FBS. In all experiments, the HA productions were expressed as ng/10^5^ cells.

The effect of 4-MU on proliferation, metabolic activity and viability were measured using Cell Proliferation ELISA BrdU Colorimetric Kit (F. Hoffmann-La Roche Ltd, Basel, Switzerland), EZ4U Assay (Biomedica Medizinproducte GmbH & Co KG, Vienna Austria) and Vybrant Cytotoxicity Assay Kit (Thermo Fisher Scientific), respectively. The detection of caspase-3 and -7 activities was performed using the Caspase-Glo 3/7 Assay System (Promega Corporation, Madison, WI, USA).

### Real-Time Polymerase Chain Reactions (RT-PCR)

The supernatants were removed and cells were washed with Dulbecco's phosphate-buffered saline solution. TRI Reagent Solution was used for the isolation of RNA from cells. The purified RNA samples were reverse transcribed by High Capacity cDNA Reverse Transcription Kit (Applied Biosystems, Thermo Fisher Scientific, Waltham). The TaqMan Gene Expression Assay (Applied Biosystems, Thermo Fisher Scientific, Waltham) was used for the detection of the expression of *HAS1*, *HAS2*, *HAS3*, *HYAL1* and *HYAL2*; glyceraldehyde 3-phosphate dehydrogenase (*GAPDH*) was used as housekeeping gene. The reactions were performed by the Real-time PCR System (BioRad, Hercules, CA, USA). Results were normalized to *GAPDH* mRNA levels by the ΔCT method.

### Statistical Analysis

Statistical analysis was performed using the STATISTICA 13 software (StatSoft Inc. Tulsa, OK, USA). Data are expressed as mean ± SD. Repeated measures analysis of variance (ANOVA) with treatment as the within-subjects factor and origin of fibroblasts (normal orbital fibroblasts, Graves’ orbitopathy fibroblasts and dermal fibroblasts) as between-subjects factor and Fisher LSD post hoc analysis were performed to evaluate the differences. The level of statistical significance was set at *P* < 0.05.

## Results

**Figure 1.**
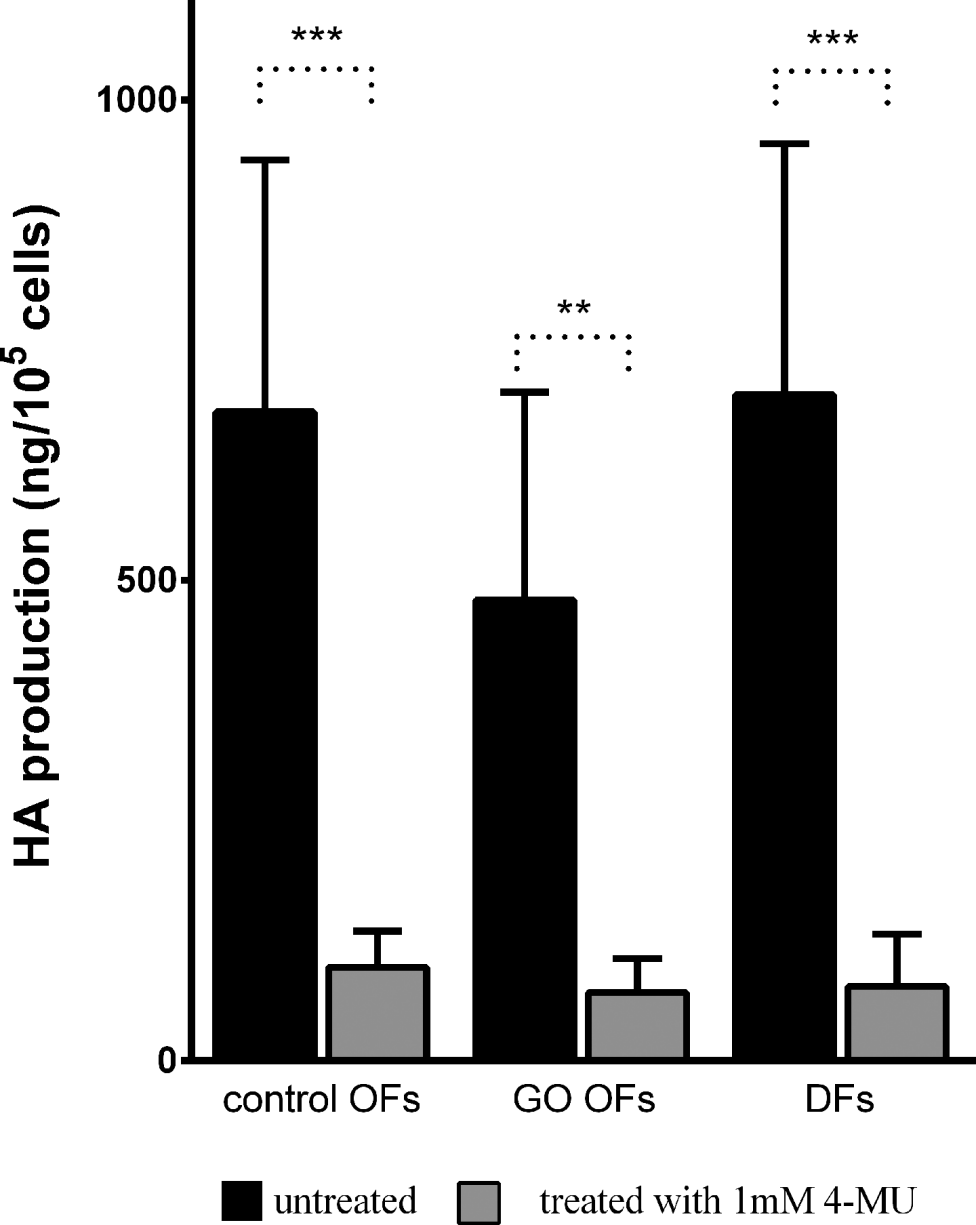
Effect of 4-MU treatment (1 mM) on hyaluronan production of control OFs, GO OFs, and DFs. Hyaluronan concentration in the supernatant was expressed in ng/10^5^ cells. Data were analyzed using repeated measures ANOVA followed by Fisher LSD post hoc test, results are shown as mean ± SD from five independent experiments. ** *P* < 0.005, *** *P* < 0.001.

The used 12 cell lines represented 3 different types of cells: control OFs (n = 4), GO OFs (n = 4), and DFs (n = 4); the amount of HA produced by these cells under normal conditions was 675 ± 262, 479 ± 217, and 693 ± 262 ng hyaluronan/10^5^ cells/24 hours, respectively (Fig. 1, dark columns). The origin of fibroblasts was not a significant factor in the HA production (control OFs vs. GO OFs: *P* = 0.138, control OFs vs. DFs: *P* = 0.884, GO OFs vs. DFs: *P* = 0.091).

**Figure 2.**
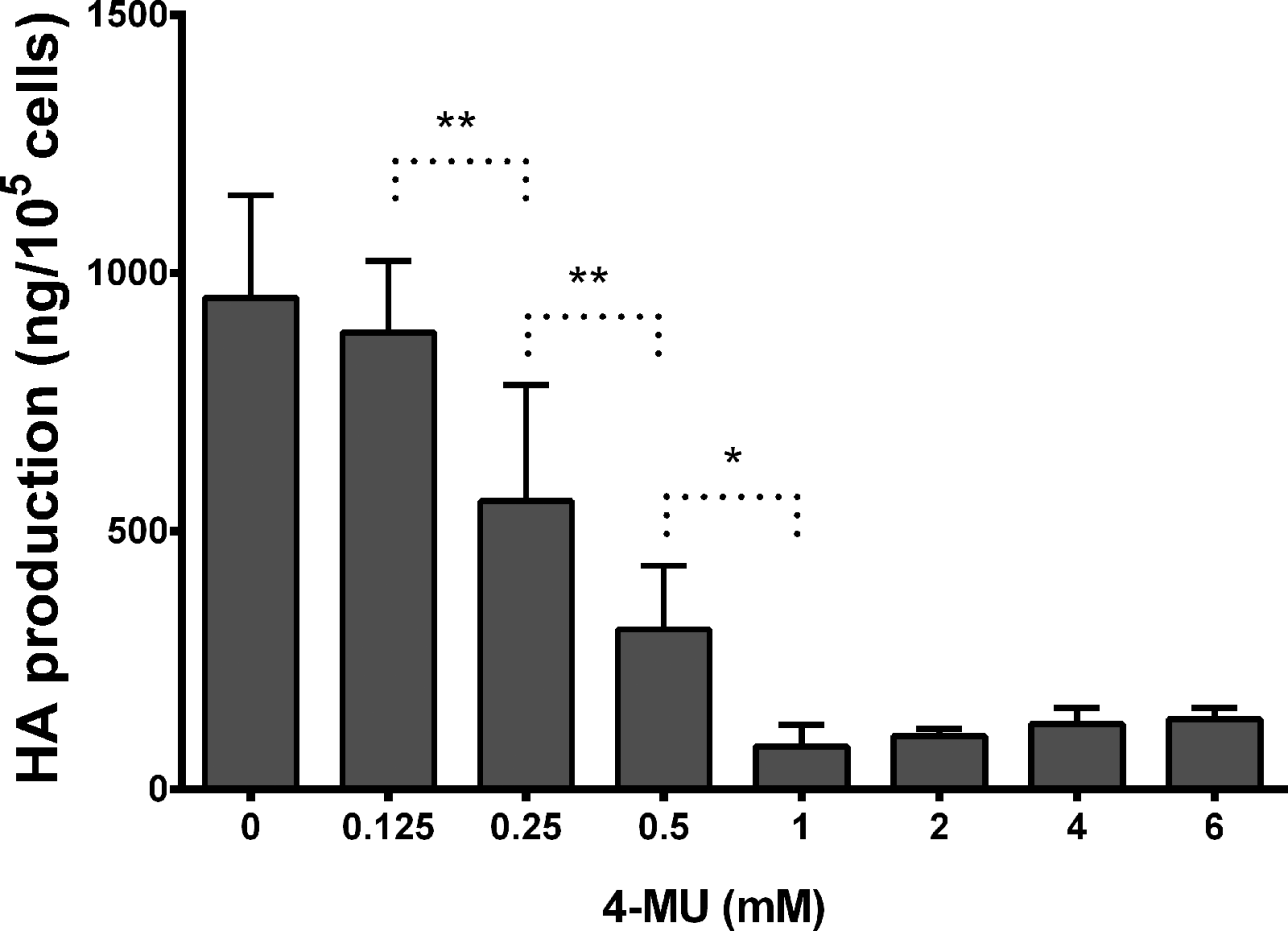
Dose-dependent influence of 4-MU treatment (0.125–6.0 mM) on hyaluronan production of OFs. Hyaluronan concentration in the supernatant was expressed in ng/10^5^ cells. Data were analyzed using repeated measures ANOVA followed by Fisher LSD post hoc test, results are shown as mean ± SD. * *P* < 0.01, ** *P* < 0.005.

HA production of the fibroblasts has been measured in the supernatants after 24-hour 4-MU treatment. The HA secreted by individual cells decreased after 4-MU treatment in a dose-dependent manner. Dose-response experiments were performed in the range of 0.125–6.0 mM in OFs. In the examined range, maximum effect has been reached at 1 mM 4-MU (Fig. 2); this concentration was used in all experiments concerning HA production and gene expressions. A strong inhibitory effect was observed in HA production when OFs and DFs were treated with 1 mM 4-MU (Fig. 1, gray columns). The inhibition was 85.3% ± 2.9% in control OFs (*P* < 0.001), 85.4% ± 2.8% in GO OFs (*P* < 0.005), and 91.3% ± 1.2% in DFs (*P* < 0.001); the 1 mM 4-MU treatment caused an average 86.7% decrease in HA production. The effectiveness of 4-MU did not depend on the origin of the fibroblasts (*P* = 0.352).

There was a similar change in the amount of pericellular HA after 24 hours’ 4-MU treatment (*P* < 0.05) in both control and GO OFs, whereas a less marked effect was seen in DFs. The inhibition was 57% ± 38% in control OFs, 58% ± 31% in GO OFs, and 29% ± 7% in DFs.

To check whether 4-MUG, the main metabolite of 4-MU, has an effect on HA synthesis, 4-MUG was added to the cell culture under the same conditions (1 mM for 24 hours). 4-MUG did not affect HA production (data not shown).

**Figure 3.**
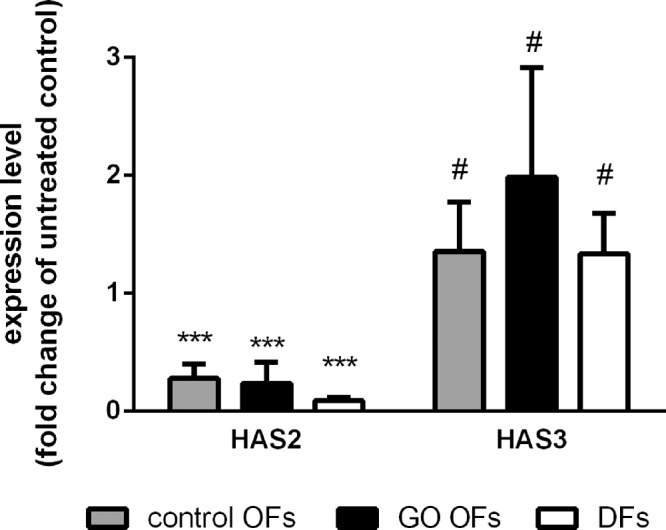
Influence of 4-MU treatment (1 mM) on *HAS2* and *HAS3* mRNA expression levels in control OFs, GO OFs, and DFs. Results adjusted for the expression of GAPDH. Data were analyzed using repeated measures ANOVA followed by the Fisher LSD post hoc test. Results are shown as fold change of untreated (mean ± SD) from 3 independent experiments. # *P* < 0.05, *** *P* < 0.001.

Next, we studied the effect of 4-MU on mRNA expression of *HAS1*, *HAS2*, *HAS3*, *HYAL1,* and *HYAL2*. *HAS1* expression was too low in most of the studied cell lines to accurately measure its basal expression. The 4-MU treatment resulted in a significant decrease in *HAS2* expression (*P* < 0.001) in all cell lines (Fig. 3), and there was no difference in the effect according to the site of origin (*P* = 0.268). The reductions in *HAS2* expression were 72% ± 12%, 76% ± 18% and 91% ± 3% for control OFs, GO OFs, and DFs, respectively. In the case of *HAS3* (Fig. 3) 4-MU treatment caused a significant increase in the expression in fibroblasts (*P* = 0.030) irrespective of the site of origin (*P* = 0.163). The increase was 136% ± 42% in control OFs, 199% ± 93% in GO OFs and 134% ± 35% in DFs compared with baseline.

**Figure 4.**
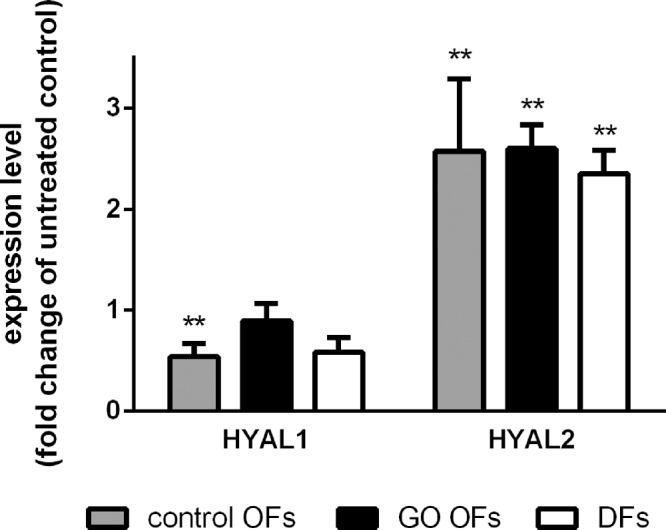
Influence of 4-MU treatment (1 mM) on *HYAL1* and *HYAL2* mRNA expression levels in control OFs, GO OFs, and DFs. Results were adjusted for the expression of GAPDH. Data were analyzed using repeated measures ANOVA followed by the Fisher LSD post hoc test, results are shown as fold change of untreated (mean ± SD) from 3 independent experiments. ** *P* < 0.005.

When hyaluronidases were studied, 4-MU treatment decreased *HYAL1* expression (*P* = 0.017), and the effect was dependent on the site of origin (*P* = 0.042) (Fig. 4). The post hoc analysis showed that the 4-MU treatment decreased *HYAL1* only in control OFs, by 45% ± 13% (*P* < 0.005). The 4-MU treatment caused a significant increase in the *HYAL2* expression (*P* < 0.0001) (Fig. 4), and this effect was not dependent on the origin of fibroblasts (*P* = 0.368). The increase was 257% ± 72% in control OFs, 260% ± 24% in GO OFs, and 235% ± 24% in DFs compared with baseline.

**Figure 5.**
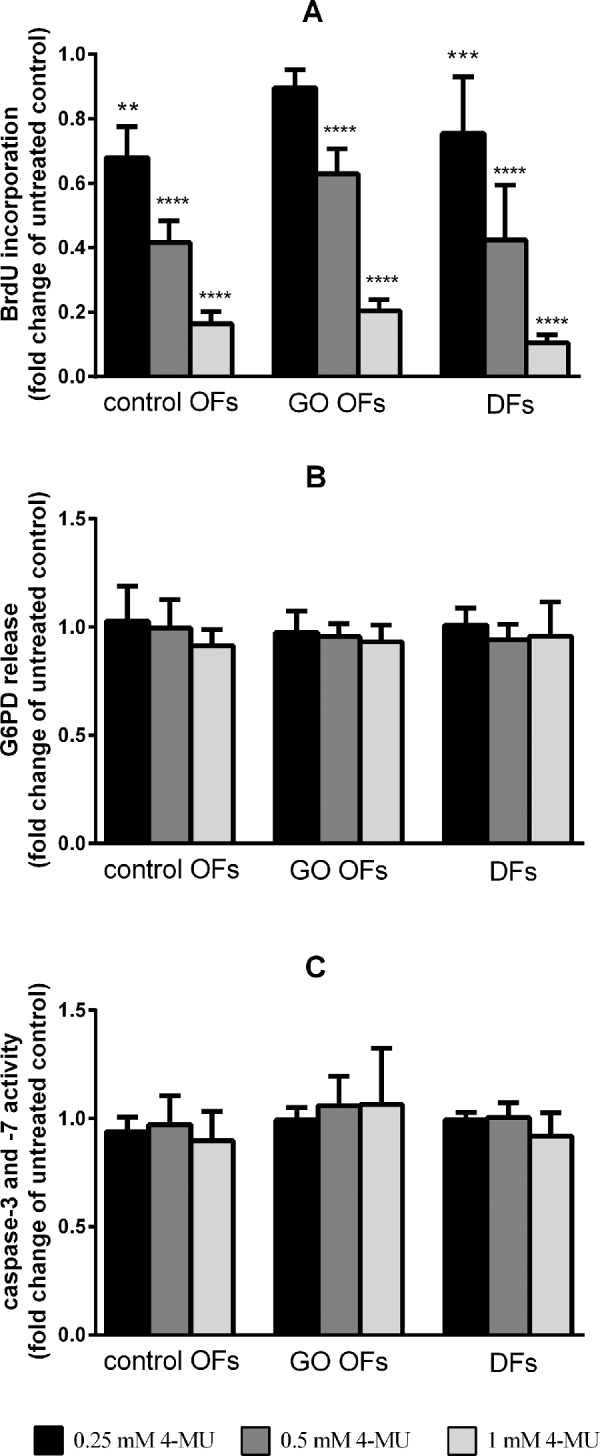
The effect of 4-MU treatment (0.25, 0.5 and 1 mM) on proliferation rate (**A**), viability (**B**), and caspase-3 and -7 activity (**C**) of control OFs, GO OFs, and DFs. Data were analyzed using repeated measures ANOVA followed by the Fisher LSD post hoc test, results are shown as fold change of untreated (mean ± SD) from 3 independent experiments. ** *P* < 0.005, *** *P* < 0.001, **** *P* < 0.0001.

Fibroblast proliferation is an important factor in the autoimmune process of GO. The proliferation potential of cultures was estimated by BrdU assay. The proliferation rate was decreased by 84% ± 4% in control OFs, 80% ± 3% in GO OFs and 90% ± 2% in DFs incubated with 1 mM 4-MU for 24 hours compared with cultures incubated in medium alone (*P* < 0.001). As shown in Figure 5A, the effect of 4-MU on the proliferation rate was dose dependent in the range of 0.25–1 mM. Both the baseline proliferation rate and the response to 4-MU were independent of the anatomical origin of fibroblasts (*P* = 0.387 and *P* = 0.259, respectively). To show that reduced cell proliferation is not the consequence of direct toxicity of 4-MU, we used a tetrazolium salt based assay first. The conversion of tetrazolium to formazan by the cells was decreased after 1 mM 4-MU treatment (*P* < 0.0001) by 29% ± 18% in control OFs, 34% ± 11% in GO OFs, and 30% ± 10% in DFs; the effect of 4-MU was not different in the fibroblasts with distinct type of origin (*P* = 0.356). It is known that over/underestimation of cell viability by tetrazolium salt–based assays could happen in the case of certain agents with modulating effects on metabolism.^21^ To avoid this bias, we completed the study with a nonmetabolic cytotoxicity assay based on measuring G6PD release from damaged/dying cells to determine the cytotoxicity of 4-MU. The ratio of damaged or dead cells in the 4-MU-treated cultures did not differ from that observed in the cultures without 4-MU in any of the studied cell lines (Fig. 5B). Although cell viability was found unchanged within 24 hours of 4-MU treatment, potential pro-apoptotic activity of 4-MU was tested performing caspase-3 and -7 activity measurement. No enhancing effect of 4-MU on executioner caspases was found within 24 hours (Fig. 5C).

**Figure 6.**
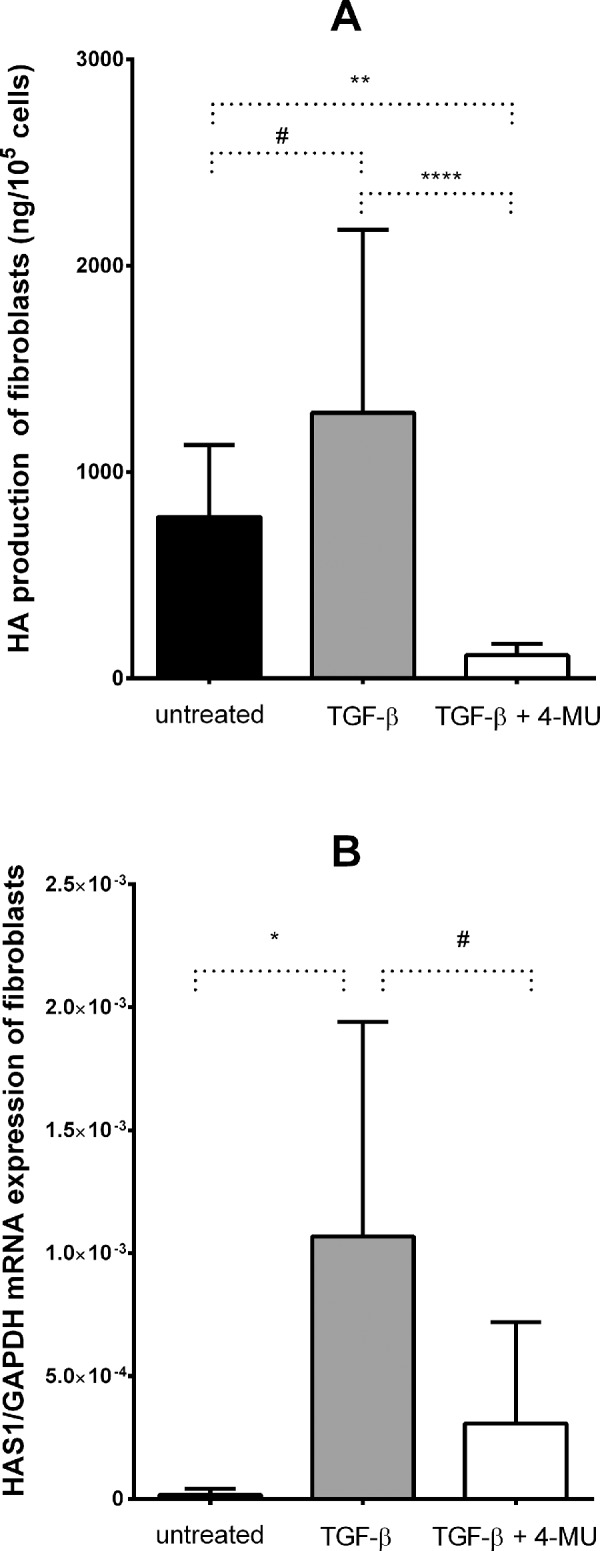
The effect of 4-MU treatment (1 mM) on TGF-β stimulated (1 ng/L) HA production (**A**) and *HAS1* expression (**B**) in the studied fibroblast lines. Data were analyzed using repeated measures ANOVA followed by Fisher LSD post hoc test, results are shown as mean ± SD from 3 independent experiments. # *P* < 0.05, * *P* < 0.01, ** *P* < 0.005, **** *P* < 0.0001.

In a recent study we found that TGF-β increased the HA synthesis of OFs and DFs through stimulating the expression of HAS1.^11^ Now we studied the effect of 4-MU on the HA synthesis of TGF-β-stimulated cells. The 4-MU decreased HA production added simultaneously with TGF-β (1 ng/mL) in every studied cell line where TGF-β alone (1 ng/mL) stimulated the HA synthesis (untreated vs. TGF-β treated: *P* = 0.036, TGF-β treated vs. TGF-β + 4-MU treated: *P* < 0.0001, untreated vs. TGF-β + 4-MU treated: *P* < 0.005) (Fig. 6A). There was no difference between fibroblasts with different type of origin in this respect (*P* = 0.242). TGF-β also stimulated *HAS1* expression (*P* < 0.01); the stimulatory effect was decreased by 4-MU (P = 0.034) (Fig. 6B). The expression of *HAS2*, *HAS3*, *HYAL1* and *HYAL2* of untreated or 4-MU treated cells did not change significantly by TGF-β treatment.

No associations were observed between any patient characteristics and hyaluronan production of the respective fibroblast line examined either in basic or in stimulated conditions.

## Discussion

HA is a major factor in the pathogenesis of GO^1^; in addition to the overproduction of HA by fibroblasts, HA may potentially retain extracellular fluid about 1000 times of its own weight,^22^ leading to edema, increased orbital pressure, and proptosis. In addition, HA may have a crucial role in the chronic autoimmune inflammation affecting retrobulbar connective tissue and external eye muscles. Any therapeutic measure diminishing local HA production can potentially reduce intraorbital pressure and could interfere with the pathogenesis of GO. 4-MU has successfully been used in animal models of autoimmunity where HA is supposed to contribute to disease pathogenesis.^16^^,^^19^^,^^20^ Our in vitro study on the characteristics of HA synthesis inhibition by 4-MU in OFs may serve as a starting point for further research on the potential beneficial effects of limiting HA production in GO.

In line with earlier studies on other cell types,^13^^–^^16^^,^^23^^,^^24^ we have found that 4-MU in the same concentration range has inhibited HA synthesis in normal and GO OFs, as well as DFs examined, even in the presence of TGF-β. HA production of the OFs originated either from GO or non-GO control orbits decreased in the same extent as it was observed in the fibroblasts with dermal origin. This inhibition during 24-hour 4-MU treatment is mainly due to the well-known mechanism that 4-MU depletes the cellular pool of the HA synthesis precursor, UDP-GlcUA.^17^ Because it is known that 4-MU treatment reduces the expression of HAS enzymes,^18^ we measured the effect of 4-MU on the transcriptional levels of several enzymes involved in HA metabolism. We found here that 4-MU greatly reduced *HAS2* mRNA expression, whereas it increased mildly the *HAS3* expression in OFs and DFs. The same effect on *HAS2* expression was described in cancer cells in which the level of *HAS3* mRNA was also reduced except for 1 cell line with very low *HAS3* expression level.^18^ We assume that the effect of 4-MU on transcription of HAS genes with very low expression levels is less consistent; *HAS2* expression was 2 orders of magnitude higher than *HAS1* and *HAS3* in the studied cells, as we described earlier.^11^ This theory is supported by our observation that significant downregulating effect of 4-MU on the expression of *HAS1* was observed in TGF-β stimulated cells where *HAS1* transcription is substantially higher (Fig. 6B). The direct effect of 4-MU on the expression of *HAS* genes is still unclear, but it was described earlier that *HAS1* and *HAS2* expressions positively correlated with the cellular level of both UDP-GlcUA and UDP–N-acetyl-glucosamine,^25^ suggesting a strong indirect effect of 4-MU due to the UDP-GlcUA depletion. No correlation was found between *HAS3* expression and intracellular UDP-sugar contents^25^ suggesting that the effect of 4-MU on *HAS3* expression is less predictable. The contribution of the 4-MU induced changes in the expression pattern of HAS enzymes to the reduced HA production is uncertain given the fact that UDP-GlcUA starvation prevented the localization of the intact HAS on the plasma membrane,^26^ where HA synthesis can occur.

Besides the HAS enzymes, 4-MU affected the expression of hyaluronidases. HYAL2 is anchored to the cell surface and generates intermediate-size HA fragments only from the high molecular weight HA (HMW-HA), whereas HYAL1 is active in the lysosome and degrades any size of HA as substrate generating very small HA fragments.^5^^,^^12^^,^^27^ Our finding of an increased *HYAL2* expression in response to 4-MU may mean acceleration of HA turnover which may have a beneficial effect for the disease process in GO. The reduction of *HYAL1* expression observed only in control OFs is unlikely to influence HA metabolism to a great extent in these cells; additional enzymes participate in the lysosomal HA degradation.^5^ We assume that the main effect through which 4-MU influences HA production in addition to its role as a competitor for UDP-GlcUA is its reducing effect on *HAS2* expression.

In previous studies on OFs the decline of HA production was coupled with the reduction of cell proliferation.^28^^,^^29^ The decreased HA synthesis by 4-MU treatment was found to be associated with a strong reduction of proliferation rate in keratinocytes and aortic smooth muscle cells,^13^^,^^24^ which has now been confirmed in our study in OFs and DFs. Inhibition of HA synthesis leads to cell cycle arrest at mitosis, just before cell rounding and detachment.^30^ A same effect of HA synthesis inhibition by 4-MU was demonstrated in osteosarcoma cells.^31^ In tumor cells 4-MU has been reported to interfere with proliferation, motility and invasion by inhibiting HA signaling events, including survival-related pathways such as the PI3K/Akt pathway by suppressing phosphorylation of Akt.^31^^–^^34^ In the aforementioned studies downregulation of the anti-apoptotic PI3K/Akt pathway by 4-MU was accompanied by induction of apoptosis.^31^^–^^33^ We did not find apoptotic effect of 4-MU in OFs and DFs within 24 hours of treatment by performing caspase-3 and -7 activity assay; further studies are needed to clarify this issue during longer treatment.

4-MU has not been found cytotoxic in the concentration required for maximal inhibitory effect on HA synthesis. Based on our results with several different cytotoxicity assays, we assume that the formazan-based metabolic assay could overestimate the effect of 4-MU on cell viability.

Because the tissue microenvironment, including its HA content contributes to the characteristics of local inflammatory processes,^35^ 4-MU in GO may have several beneficial effects in the initiation and the course of the disease. The 4-MU treatment interfered with cell-cell interaction required for antigen presentation^36^ and inhibited T-cell proliferation.^37^ 4-MU has been found to be beneficial in animal models of autoimmune diseases associated with increased regulatory T-cell numbers, which play a critical role in the protection from autoimmunity and fibrotic conditions.^19^^,^^20^ In addition to limiting HA synthesis and fibroblast proliferation, another potential positive impact of 4-MU may be its modulating effect on adipogenesis of OFs, which is an important step leading to proptosis in the pathomechanism of GO.^38^ Adipogenesis in OFs was accompanied by HA accumulation and increased *HAS2* expression.^39^ 4-MU has been described as an inhibitor of adipogenic differentiation in 3T3-L1 fibroblasts^40^ and reduced fat pad weight and adipocyte size in vivo.^41^

However, some questions arise about the use of 4-MU as a medication. It is unclear whether, and to what extent the effect of 4-MU is modulated by the concomitant administration of other drugs that are metabolized via the UDP-glucuronyltransferases–mediated pathway. Another doubt is the low bioavailability and short half-life of 4-MU; in a recent study its main metabolite 4-MUG was found to contribute to the bioactivity of 4-MU both in vitro and in vivo via conversion to 4-MU.^42^ This conversion was observed in cell cultures after 48–72 hours^42^; this may explain why the 24-hour 4-MUG treatment in our study was not sufficient to exert any effect on OFs and DFs.

One potential limitation of our study is its in vitro nature. It is not clear to what extent our findings can be translated to the human orbit, where a more complex local environment, interaction of immune, endocrine and local factors, including increased tissue pressure, contribute to disease pathogenesis. Nevertheless, any of these factors are potential targets for intervention.

We have shown here that 4-MU interferes with fibroblast activity in at least 2 different ways, by diminishing proliferation and inhibiting HA synthesis. Further studies in animal models of GO and a clinical trial could provide further information on the feasibility of 4-MU as a therapeutic agent in this disease.
